# Supplementary material for: ‘Experiences of patients and their informal caregivers with cognitive stimulation programs for dementia: A qualitative systematic review protocol’
Source: PLoS One. 2023 Jun 28;18(6):e0287851. doi: 10.1371/journal.pone.0287851 (PMC10306174; doi:10.1371/journal.pone.0287851)
Supplement: S1 File — (DOCX) [file pone.0287851.s002.docx]

**S2 Table***-* MEDLINE (Ovid) Search strategy

| Ovid MEDLINE(R) ALL <1946 to January 12, 2023>  1 exp Dementia/ 199007  2 (dementia or alzheimer* or vascular dementia or mixed dementia or major neurocognitive disorder or frontotemporal dementia or hungtington* or lewy body or parkinson's disease dementia).mp. [mp=title, book title, abstract, original title, name of substance word, subject heading word, floating sub-heading word, keyword heading word, organism supplementary concept word, protocol supplementary concept word, rare disease supplementary concept word, unique identifier, synonyms] 291863  3 1 or 2 310956  4 exp Caregivers/ 48301  5 (caregiv* or carer* or spouse or family or relative* or care giv*).mp. [mp=title, book title, abstract, original title, name of substance word, subject heading word, floating sub-heading word, keyword heading word, organism supplementary concept word, protocol supplementary concept word, rare disease supplementary concept word, unique identifier, synonyms] 2765540  6 exp Family/ 361635  7 exp Spouses/ 11453  8 exp Adult Children/ 1796  9 exp Fathers/ 10690  10 exp Mothers/ 53930  11 4 or 5 or 6 or 7 or 8 or 9 or 10 2934451  12 (cognitive stimulation or cognitive stimulation therapy or cst).mp. [mp=title, book title, abstract, original title, name of substance word, subject heading word, floating sub-heading word, keyword heading word, organism supplementary concept word, protocol supplementary concept word, rare disease supplementary concept word, unique identifier, synonyms] 6483  13 exp Qualitative Research/ 79023  14 (qualitative or perspective* or view* or experience* or attitude* or impact).mp. [mp=title, book title, abstract, original title, name of substance word, subject heading word, floating sub-heading word, keyword heading word, organism supplementary concept word, protocol supplementary concept word, rare disease supplementary concept word, unique identifier, synonyms] 3727891  15 13 or 14 3727960  16 3 or 11 3196934  17 12 and 15 and 16 330 |
| --- |
|  |
