# Supplementary material for: ‘Experiences of patients and their informal caregivers with cognitive stimulation programs for dementia: A qualitative systematic review protocol’
Source: PLoS One. 2023 Jun 28;18(6):e0287851. doi: 10.1371/journal.pone.0287851 (PMC10306174; doi:10.1371/journal.pone.0287851)
Supplement: Attachment — Submitted filename: Response to Reviewers.docx [file pone.0287851.s004.docx]

From

Simone Ryan

Occupational therapy graduate

University of Galway

To

Dr Michael Gilbert McCaul

Academic Editor

PLOS ONE

Sub: Submission of the revised manuscript - PONE-D-23-01290.

Dear Dr McCaul,

We are pleased to submit our revised qualitative systematic review protocol titled, "Experiences of patients and their informal caregivers with cognitive stimulation programs for dementia: a qualitative systematic review protocol" to your esteemed journal.

Many thanks for the constructive feedback from the reviewers. The manuscript has been revised significantly in response to all the comments. In order to assist the review process, a detailed response is attached below to each statement/comment made by the reviewers. We believe that the revised manuscript meets the requirements for publication.

Thanking you

With best regards,

Simone Ryan

**AUTHORS’ RESPONSE TO THE REVIEWERS’ COMMENTS**

| **COMMENTS FROM REVIEWER 1** | **Authors’ Responses** |
| --- | --- |
| 1. I suggest referring to 'caregiver(s)' as 'informal caregiver(s)' throughout the protocol (including the title). While this clarification is provided in the 'inclusion criteria' section of the manuscript, I believe it would be easier for the reader if you clarified this earlier in the manuscript. | Thank you for the suggestion. The revised manuscript replaces the word caregiver(s) with informal caregiver(s) throughout the revised manuscript. |
| *2. Do you plan to conduct any reflexivity exercises with the team who will be synthesising the data?* | Thanks for the suggestion. We agree that reflexivity is an important factor that ensures rigour in qualitative systematic reviews. Not including this in our original manuscript is an oversight. We have now included a paragraph on reflexivity under the methods heading of the manuscript.  The revised version reads as follows:  It is important to consider the reviewers' assumptions and preconceptions regarding the phenomenon of interest in qualitative systematic reviews, as well as the possible influence these assumptions may have on the review process. The reviewers will therefore make conscious efforts to minimise their preconceived ideas and assumptions from influencing the review by writing memos and reflecting upon these during data collection, analysis and writing up the review. During the memo-writing process, methodological notes explaining procedural decisions and observational comments will be written to describe and explore the reviewer's feelings at different stages. |
| 3. *How do you plan to synthesise findings from patients and caregivers? Where possible, will you plan on separating the findings from these two participant groups? Obviously this will be dependent on how individual articles have analysed and reported their findings, but I believe it would be worthwhile outlining how you plan to analyse findings from the two participant groups.* | Thanks for your suggestion. We have now rewritten the data synthesis section providing more detail that answers your question.  The revised version reads as follows:  Data will be disaggregated for people with dementia and informal caregivers where possible. However, since disaggregation of data significantly depends on the availability of individual articles on the topic area and how they analyse and report their findings, such a decision would be made as part of meta-aggregation in accordance with the JBI methodology for systematic reviews of qualitative data. |
| *4. I think the discussion could be expanded on by reference to national/international dementia care guidelines and how your findings will complement these guidelines. In recent years there has been an increased focus on dementia care, with an associated increase in the number of clinical practice guidelines, and it would be interesting to consider how your review may contribute to our understanding of current best practice.* | Thanks for the suggestion. We have now incorporated this information in our revised manuscript.  The revised version reads as follows:  "The National Institute for Health and Care Excellence (NICE) guidelines for the assessment, management and support of people living with dementia [34] recommend CST, a common but specific type of cognitive stimulation intervention, be provided to people with mild-to-moderate dementia. While there are no explicit and formal mentions or recommendations for other types of cognitive stimulation interventions in existing clinical practice guidelines, findings from the proposed review of patients' and their informal caregivers' experiences with all types of cognitive stimulation are likely to offer valuable information that can influence best practice guidelines and recommendations with appropriate suggestions regarding the delivery of these interventions." |
|  |  |
| **COMMENTS FROM REVIEWER 2** | **Authors’ Responses** |
| *16-21: In the first two lines of the abstract, the author tends to foreground the caregivers. I suggest that the voices of those with dementia and the problem statement are highlighted. The rationale for the inclusion of caregivers is not needed in the abstract.* | We understand and agree with the reviewer's view that our abstract from our original manuscript may have unintentionally kept the caregivers in the foreground. Therefore, considering the reviewer's suggestion, we have revised this sentence and rewritten this section in our revised manuscript.  The revised version reads as follows:  "The patient’s experience of a complex intervention is unique and often determines its effectiveness. This proposed qualitative systematic review aims to comprehensively synthesise the experiences of individuals with dementia and their informal caregivers who have participated in cognitive stimulation programs, identifying perceived benefits, challenges, barriers, and facilitators to this approach to intervention." |
| *124-134: The description of existing reviews on the similar topic needs to be edited in improve readability. The first review is reported as broad and the second as more specific but neither as comprehensive. Perhaps each review can be critically reported and how the proposed review fills an identified gap.* | Following the reviewer's suggestions, we have rewritten the original manuscript's content presented in lines 122-134.  The revised version reads as follows:  "The scope of Tuomikoski et al.’s review, which examined the experiences of people with progressive memory disorders who have participated in non-pharmacological interventions, was broader than the proposed review since it evaluated cognitive stimulation alongside other non-pharmacological interventions, such as exercise programs and music therapy [21]. Gibbor et al., on the other hand, focused their review on a specific type of cognitive stimulation intervention: CST and its variations, excluding other forms of cognitive stimulation interventions [20]. While both these reviews are rigorous, of high quality, and critical in synthesising the experiences of their respective interventions of interest, neither review gives a comprehensive summary of the experience of participating in all types of cognitive stimulation interventions. The inclusion of all types of cognitive stimulation intervention programs, such as non-manualised, individual, or multi-component programs, as well as CST, would provide a more comprehensive meta-synthesis of the experience of participating in a cognitive stimulation intervention. |
| 259: *Although the methods are well designed, the data synthesis for the meta-aggregation needs some refinement. Specifically, authors should consider providing information on the following.*  *Who will conduct the synthesis in JBI SUMARI?*  *Where pooling is not possible who will write the narrative synthesis?*  *How will credible findings be determined?*  *Will data be disaggregated for people with dementia and caregivers?*  *How will the data be presented?* | Thanks for your suggestion. We have now rewritten the data synthesis section providing more detail and answering all your questions.  The revised version reads as follows, addressing every single question individually:  **-Who will conduct the synthesis in JBI SUMARI?**  The two independent reviewers (SR and MC) will initially review all the findings and perform synthesis or aggregation of findings based on similarities in their meaning. Where disagreements may arise, these will be resolved through discussion or involving an additional reviewer (OB).  **-Where pooling is not possible who will write the narrative synthesis?**  Where textual pooling is not possible, the findings will be presented in narrative format by the same two independent reviewers and confirmed by the review team.  **How will credible findings be determined?**  Each extracted finding and its accompanying illustrations will be rated by two independent reviewers (SR and MC) based on their perceptions of how well each illustration supports the specific finding associated with it by utilising three levels of credibility in accordance with the JBI methodology for systematic reviews of qualitative data. The three levels of credibility are unequivocal, credible and not supported; where "unequivocal" refers to a finding that is clearly related, supported, and accompanied by illustrations beyond a reasonable doubt, "credible" refers to a finding accompanied by an illustration but the illustration only vaguely demonstrates an association with the finding and "not supported" refers to a finding with no illustration to support it or the provided illustrations are entirely unrelated to the finding.  **-Will data be disaggregated for people with dementia and caregivers?**  Data will be disaggregated for people with dementia and informal caregivers where possible. However, since disaggregation of data significantly depends on the availability of individual articles on the topic area and how they analyse and report their findings, such a decision would be made as part of meta-aggregation in accordance with the JBI methodology for systematic reviews of qualitative data.  **How will the data be presented?**  Again, the review findings will be presented per the guidelines provided in the JBI Manual for Evidence Synthesis. There will be a meta-aggregative schematic/overview flow chart and a meta-aggregative schematic table with relevant narratives to explain the categories and synthesised findings. |
| *286: The review aim is more descriptive in the discussion that in the abstract in line 19 and in the introduction in line 137. These should be aligned.* | Thank you for pointing out our lapse and oversight in not ensuring consistency. Consistency in the review aim/objective is now ensured throughout the revised manuscript making necessary changes to lines 19 and 137 of the original manuscript. |
