# Supplementary material for: ‘Experiences of patients and their informal caregivers with cognitive stimulation programs for dementia: A qualitative systematic review protocol’
Source: PLoS One. 2023 Jun 28;18(6):e0287851. doi: 10.1371/journal.pone.0287851 (PMC10306174; doi:10.1371/journal.pone.0287851)
Supplement: Attachment — Submitted filename: Reviewer Comments Dementia Intervention Protocol PLOS May 2023.docx [file pone.0287851.s003.docx]

In review of the protocol ‘Experiences of patients and their informal caregivers with cognitive stimulation programs for dementia: a qualitative systematic review protocol’, I have recommended minor revisions. The proposed qualitative evidence synthesis (QES) will make a strong contribution to the literature. Herewith my minor suggestions for consideration.

Abstract:

16-21: In the first two lines of the abstract, the author tends to foreground the caregivers. I suggest that the voices of those with dementia and the problem statement are highlighted. The rationale for the inclusion of caregivers is not needed in the abstract.

Introduction:

124-134: The description of existing reviews on the similar topic needs to be edited in improve readability. The first review is reported as broad and the second as more specific but neither as comprehensive. Perhaps each review can be critically reported and how the proposed review fills an identified gap.

Methods:

259: Although the methods are well designed, the data synthesis for the meta-aggregation needs some refinement. Specifically, authors should consider providing information on the following.

Who will conduct the synthesis in JBI SUMARI?

Where pooling is not possible who will write the narrative synthesis?

How will credible findings be determined?

Will data be disaggregated for people with dementia and caregivers?

How will the data be presented?

Discussion:

286: The review aim is more descriptive in the discussion that in the abstract in line 19 and in the introduction in line 137. These should be aligned.

Thank you for the opportunity to review this work and I look forward to seeing the findings of the review.
